# Gut Modulators Alter BDNF/Cortisol Axis in Severe Obesity: Additional Secondary Outcomes from a Triple-Blind Randomized Trial

**DOI:** 10.3390/life16060924

**Published:** 2026-05-31

**Authors:** Dayanne da Silva Borges, Ricardo Fernandes, Barbara Beatriz Philippi Martins, Scheila Iria Kraus, Erasmo Benicio Santos de Moraes Trindade, Adair Roberto Soares Santos

**Affiliations:** 1 Post-Graduate Program in Neuroscience, Center of Biological Sciences, Federal University of Santa Catarina, Florianopolis 88040-535, SC, Brazil; baka.pm@gmail.com (B.B.P.M.); scheilaks@gmail.com (S.I.K.); 2 Laboratory of Neurobiology of Pain and Inflammation, Department of Physiological Sciences, Center of Biological Sciences, Federal University of Santa Catarina, Florianopolis 88040-535, SC, Brazil; 3 Post-Graduate Program in Nutrition, Research Group on Immunonutrition and Metabolism, Center of Health Sciences, Federal University of Santa Catarina, Florianopolis 88040-535, SC, Brazil; erasmotrindade@gmail.com; 4 Faculty of Health Sciences, Federal University of Grande Dourados, Dourados 79804-970, MS, Brazil; ricardontr@gmail.com

**Keywords:** obesity, depression, anxiety, prebiotic, synbiotic

## Abstract

The role of the gut–brain axis is crucial in maintaining homeostasis and regulating neural, hormonal, and immunological activity. This study aimed to evaluate the effects of prebiotics or synbiotics on serum markers related to emotional disorders in individuals with morbid obesity in a triple-blind, randomized trial. The sample consisted of 22 subjects (16 women and 6 men) with a mean age of 41.8 ± 8.5 years and a mean BMI of 47.7 ± 6.8 kg/m^2^. Serum BDNF concentrations decreased significantly after 30 days of prebiotic supplementation (*p* = 0.017). Serum cortisol concentrations increased in all groups between the evaluated time points, with the increase being statistically significant in the synbiotic-supplemented group (*p* = 0.028). Serum TNF-α concentrations increased significantly after 30 days of prebiotic supplementation when compared to the group’s baseline (*p* = 0.035); however, this variation did not produce a significant difference between the groups evaluated after 30 days of supplementation. The results suggest that a chronic, low-grade inflammatory state may be related to the neuroendocrine changes present in emotional disorders, but the absence of significant results in the primary outcomes is possibly due to severe underpowering. Findings from additional secondary outcomes are hypothesis-generating only, requiring confirmation in adequately powered trials.

## 1. Introduction

Obesity is characterized by a metabolic disorder that generates and maintains a state of chronic stress, which is considered a risk factor for emotional disorders (e.g., depression and anxiety) [1].

Depression and anxiety increase the release of corticotropin-releasing hormone (CRH) release, with a consequent increase in adrenocorticotropic hormone (ACTH) and cortisol release. In this situation, reduced glucocorticoid receptor activity precludes negative cortisol feedback over the hypothalamus while maintaining hyperactivity of the hypothalamic–pituitary–adrenal (HPA) axis [2,3,4].

Additionally, hypercortisolemia stimulates the hypothalamus to increase the release of thyrotropin-releasing hormone (TRH), followed by increased thyrotropin (TSH) release from the pituitary gland, with the consequent stimulation of the thyroid gland and increased release of thyroxine (T4). Concomitantly, increased cortisol release can negatively affect the conversion of T4 to triiodothyronine (T3), thus compromising the synthesis of serotonin (5-HT) and noradrenaline (NE) [2,3].

The synthesis of these neurotransmitters also depends on B-complex vitamins, such as B12 and folic acid, which are often reduced in individuals with depression, anxiety, and obesity [5,6]. In these situations, an increase in serum parathyroid hormone (PTH) concentrations is also observed, which in depression and anxiety may be related to increased HPA-axis activity and consequent parathyroid gland stimulation, as well as oxidative stress and vitamin D deficiency, which are also evidenced in individuals with obesity [7,8].

Currently, research indicates a strong relationship between intestinal dysbiosis and obesity, depression, and anxiety due to the increased production and systemic release of inflammatory mediators such as interleukins and transcription factors [9,10,11]. Furthermore, the sustained increase in serum concentrations of inflammatory mediators leads to chronic inflammation in the production and release of hormones such as cortisol, leptin and ghrelin, as well as contributing to vitamin deficiency. Chronic systemic inflammation and hypercortisolemia are associated with hippocampal atrophy, and with resistance to the hormones leptin and ghrelin, maintaining high serum concentrations of these hormones and fueling the release of inflammatory mediators [1,3,12,13]. Hippocampal dysfunction also promotes reduced release of brain-derived neurotrophic factor (BDNF), as evidenced in individuals with depression and anxiety. Concomitantly, B-vitamin deficiency and chronic systemic inflammation are also involved in altering blood–brain barrier (BBB) permeability, with a consequent low-grade chronic neuroinflammation related to increased CRH release and BDNF reduction [1,3,14,15,16].

Therefore, there is an essential interrelation between the gastrointestinal tract and the brain that maintains homeostasis and regulates neural, hormonal, and immune activity establishing a microbiome-gut–brain axis [17], in which the gut microbiota acts as a mediator of gut–brain communication [18]. Given the above, this study aims to evaluate the effects of prebiotic or synbiotic supplementation on serum markers related to depression and anxiety in individuals with morbid obesity.

## 2. Materials and Methods

This study is a randomized, placebo-controlled, triple-blind clinical trial conducted at the Polidoro Ernani de São Thiago University Hospital of the Federal University of Santa Catarina (HU/UFSC), Florianópolis, SC. The study sample consisted of adult individuals with morbid obesity (BMI ≥ 40.0 kg/m^2^) referred for an initial consultation at the bariatric surgery outpatient clinic of the HU/UFSC. The protocol for this study follows the precepts set out in the Declaration of Helsinki [19] and National Health Council Resolution No. 466 of 2012 [20]. The project was approved by the UFSC Human Research Ethics Committee under number 1.340.253 and was registered on the clinical trial registration *platform ClinicalTrials.gov* (http://www.clinicaltrials.gov/) under the identifier NCT02660333. This manuscript was prepared following the CONSORT (Consolidated Standards of Reporting Trials) statement [21] (Supplementary File S1) and presents results from primary outcomes (hs-CRP, IL-1β, IL-6, TNF-α) and additional secondary outcomes (BDNF, ACTH, cortisol, TSH, PTH and vitamin D) added during the blinded collection phase (month 20/26), prior to unblinding. A protocol amendment was registered (NCT02660333).

The dosage and supplementation duration were determined based on a systematic review published by our research group [22], which found that in populations with obesity, studies testing prebiotic interventions used doses ranging from 5.5 to 16 g/day, while studies testing synbiotics used 1.0 to 8.4 g/day of prebiotic combined with probiotics at 2.7 × 10^8^ to 12 × 10^9^ CFU/day. Intervention durations varied from 14 to 90 days for prebiotics and 42 to 196 days for synbiotics. Based on this systematic review, and considering the healthcare service routine where our sample was recruited as well as the available laboratory logistics, we determined the treatment duration and intervention doses.

For the inclusion criteria, adult subjects (18–60 years old) of both sexes with BMI ≥ 40.0 kg/m^2^ were selected. Subjects were excluded if they presented with previous gastrointestinal diseases; food intolerances and/or allergies; alcohol and/or drug addiction; use of anti-inflammatory, antibiotic, or immunosuppressive drugs up to three months prior to the study; regular use of laxatives, opioid narcotic analgesics and appetite suppressants; current or prior use (up to one month) of prebiotics, probiotics, synbiotics or products enriched with these ingredients; intolerance to prebiotics, probiotics, or synbiotics; following a diet for weight loss or gain in the last three months; pregnancy or lactation; currently following unusual diets (e.g., vegetarian, macrobiotic, paleolithic) and tobacco use.

All participants received standardized clarification on nutritional treatment for weight loss and guidance during the supplementation period to avoid intense physical activity; consuming alcoholic beverages and eating foods enriched with prebiotics, probiotics, or synbiotics.

The study consisted of two experimental time points: baseline—the moment of the first outpatient consultation and the beginning of prebiotic, synbiotic, or placebo supplementation; and endline—the moment after 30 days of the first outpatient consultation and the completion of prebiotic, synbiotic or placebo supplementation use. The researchers and collaborators maintained face-to-face contact with the individuals under analysis (when they visited the HU/UFSC) or by telephone call once a week to record adherence to treatment and to provide appropriate care support when needed.

Participants were randomly assigned to one of the treatment groups (G1—control group that received placebo; G2—group that received prebiotic; and G3—group that received synbiotic), using a randomization list generated by a computer program consisting of randomly interchanged blocks of three patients each. Thereafter, the treatment groups were replaced by random three-digit numeric codes also generated by a computer program. This step was performed by a researcher not involved in the study; the researcher who recruited and tracked participants only had access to the list containing randomization blocks and numeric codes, thus ensuring allocation concealment. Supplements and placebo were pre-packaged in opaque sachets and closed by the supplier with random codes, maintaining identity in physical appearance and sensory characteristics (taste and color). Supplement identification codes were revealed by the supplier company only after statistical analysis of the study data, characterizing the trial as triple blind.

### 2.1. Characterization of Nutritional Supplements

The prebiotic consisted of fructooligosaccharides (FiberFOS^®^—Invictus Farma Nutrição, Group FQM, Rio de Janeiro, Brazil), packaged in 6 g sachets. The synbiotic consisted of fructooligosaccharides and probiotics (*Lactobacillus paracasei* LPC-37 10^9^ CFU; *Lactobacillus rhamnosus* HN001 10^9^ CFU; *Lactobacillus acidophilus* NCFM 10^9^ CFU; *Bifidobacterium lactis* HN019 10^9^ UFC) (Simbioflora^®^—Invictus Farma Nutrição, Group FQM, Rio de Janeiro, Brazil), packaged in 6 g sachets. The placebo consisted of maltodextrin, packaged in 6 g sachets identical to the intervention supplements.

Subjects were instructed to consume two sachets per day (12 g/day) for 30 days at different times: one sachet (6 g) to be consumed while fasting and one sachet (6 g) between two meals. Each sachet was to be diluted in 100 mL of room-temperature water until completely dissolved. Participants missing ≥2 consecutive days of supplementation were discontinued, based on David et al. [23], who demonstrated significant microbiota composition changes after just two days of dietary intervention. Participants recorded daily supplement consumption in specific log forms returned at the study endpoint. Additionally, researchers conducted weekly phone monitoring to track adherence and monitor adverse events.

### 2.2. Characterization of Subjects and Clinical Data Collection

Subjects participating in the study were characterized at baseline using personal and clinical data. To assess nutritional status, anthropometric measurements of weight, height and waist circumference (WC) were performed by trained professionals, following techniques proposed by the World Health Organization (WHO) in 1995 and 2008 [24,25]. The Body Mass Index (BMI) was calculated and classified according to the cutoff points defined by the WHO [25].

The recorded clinical parameters included associated comorbidities, drugs used, gastrointestinal changes, the presence of constipation, and the consistency, and shape of stools. Additionally, the use of vitamin and mineral supplements, practice of physical activity and characteristics of the menstrual period were verified through surveys conducted at baseline and endline. To determine the presence of constipation, the diagnostic criteria of Rome IV were used [26]. To assess the consistency and shape of the stool, the Bristol Stool Form Scale criteria were used [27].

### 2.3. Biological Sampling and Biochemical Analysis

For laboratory analysis, peripheral venous blood samples were collected at baseline and endline by a trained professional according to standard technique. Samples were always collected in the morning before 10 a.m. after an 8 to 10 h fast. From the collected samples, plasma and serum were separated for analysis as described below:

- BDNF was determined in plasma by the enzyme-linked immunosorbent assay (ELISA) method (R&D Systems^®^, a Bio-Techne brand, Minneapolis, MN, USA);
- ACTH and cortisol were determined in plasma by the microparticle chemiluminescence immunoassay method (ACTH: CLIA Immulite 2000 XPi^®^, Siemens Healthcare Diagnostics Inc., Newark, DE, USA; Cortisol: CLIA Centaur XP^®^, Siemens Healthcare Diagnostics Inc., Newark, DE, USA);
- TSH, PTH, 25-hydroxyvitamin D (25OH), vitamin B12, and folic acid were determined in serum by the microparticle chemiluminescence immunoassay method (CMIA Architect^®^, ABBOTT Laboratories, Abbott Park, IL, USA);
- High-sensitivity C-reactive protein (hs-CRP) was determined in serum by the nephelometry method (BN II^®^, Siemens Healthcare Diagnostics Inc., Newark, DE, USA);
- IL-1β, IL-6, and TNF-α were determined in plasma by the ELISA method (BD OptEIA^TM®^, BD Biosciences, San Jose, CA, USA).

### 2.4. Statistical Analysis

This exploratory analysis tested multiple biomarkers, including secondary outcomes added during blinded data collection. No multiple testing correction was applied to preserve potential signals. All reported *p*-values are nominal/uncorrected. To calculate the sample size, a study power of 80%, a confidence interval of 95%, and a 10% increase to account for possible follow-up losses were considered. The calculation was performed based on IL-6 values (a primary outcome), resulting in a minimum sample size of 54 subjects per group. A per-protocol analysis was selected due to: (1) the short intervention duration minimizing attrition bias; (2) stringent confounder exclusion maintaining internal validity; and (3) confirmed baseline comparability across randomized groups. Statistical analysis was performed using STATA^®^ version 13.0 (StataCorp, College Satation, TX, USA).

Continuous variables were synthesized using two single measurements per group: mean and standard deviation for symmetrical distributions, and median and interquartile range for asymmetrical distributions. In contrast, categorical variables were described as frequencies and percentages within the established groups. To evaluate data distribution, the Shapiro–Wilk, Skewness, Kurtosis and Coefficient of Variation normality tests were applied.

For continuous variables, comparisons between groups (control and supplemented) were performed using one-way ANOVA (parametric data) or the Kruskal–Wallis test (nonparametric data). Tukey’s (parametric data) and Mann–Whitney U (nonparametric data) tests were used for post hoc comparisons. For comparisons within the same group between baseline and post-intervention, the Wilcoxon signed-rank test (nonparametric data) was applied.

For categorical variables, comparisons between groups (control and supplemented) were performed using Fisher’s exact test. Correlation analysis was performed using Spearman correlation (nonparametric data). For all tests, a significance level of 95% (*p* < 0.05) was adopted.

## 3. Results

**Figure 1:**
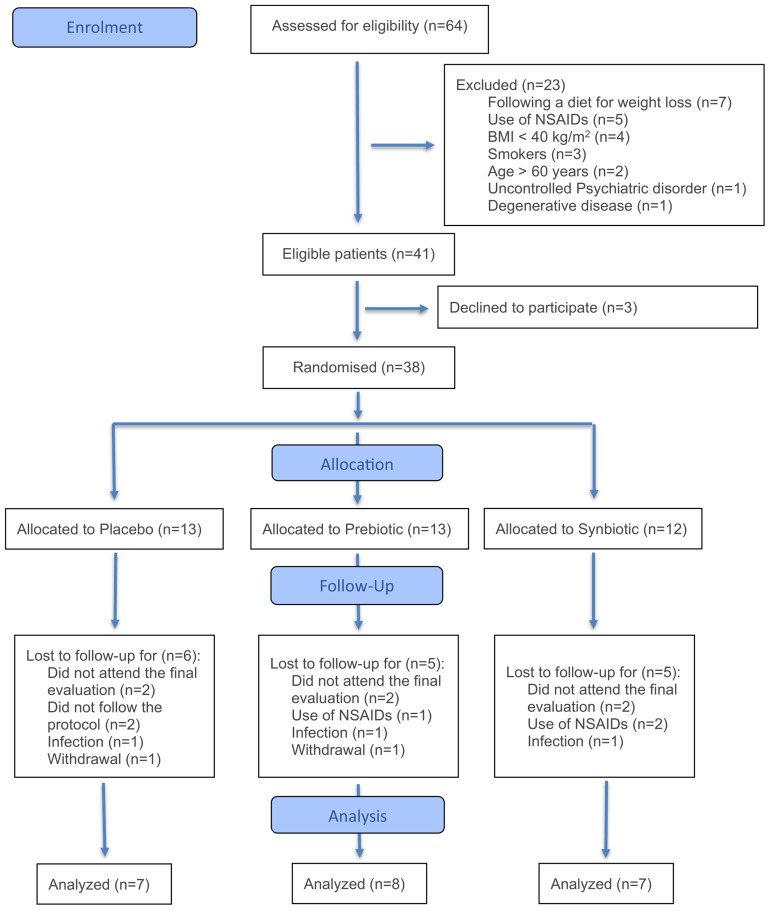
Flowchart of participant recruitment and study sample selection. NSAIDs: nonsteroidal anti-inflammatory drugs (e.g., ibuprofen, tenoxicam or acetylsalicylic acid). Source: Adapted from Hopewell et al. [21].

Between January 2016 and February 2018, 64 subjects underwent eligibility screening. Of these, 23 subjects did not meet the selection criteria, as shown in Figure 1.

Of the 41 subjects considered eligible, 3 subjects refused to participate in the study. These individuals were male, aged between 31 and 38 years old, and had a BMI between 41.1 and 59.8 kg/m^2^. Therefore, 38 subjects were randomized into the three treatment groups (placebo, prebiotic, or synbiotic). During the supplementation period, 16 subjects did not complete the study and are presented as “Follow-up Losses” in Figure 1. Of these individuals, only 8 represented true follow-up losses (scheduling issues, *n* = 6; withdrawal *n* = 2). Scheduling dropouts were contacted by phone and confirmed no adverse events related to supplementation, thereby minimizing outcome-related attrition bias. The remaining 8 subjects were proactively discontinued for protocol violations to preserve internal validity (use of nonsteroidal anti-inflammatory drugs (NSAIDs), *n* = 3; infections, *n* = 3; and non-compliance, *n* = 2). The infections that led to discontinuation involved the upper respiratory tract, and the use of NSAIDs was due to pain in the lower limbs and lumbar region. In the end, 22 subjects completed the study: 7 subjects in the placebo group; 8 subjects in the prebiotic group and 7 subjects in the synbiotic group. The number of individuals in the final sample of this study was not sufficient to identify significant differences in the primary outcomes, according to the sample size calculation. The complete sample recruitment and selection flowchart is shown in Figure 1.

The sample consisted of 16 women and 6 men, with a mean age of 41.8 ± 8.5 years old and a mean BMI of 47.7 ± 6.8 kg/m^2^. At baseline, there were no significant differences between the groups in terms of age, gender, nutritional status, and clinical parameters, comorbidities, or medications for continuous use (Table 1).

### Biochemical Variations Between Study Moments

**Figure 2:**
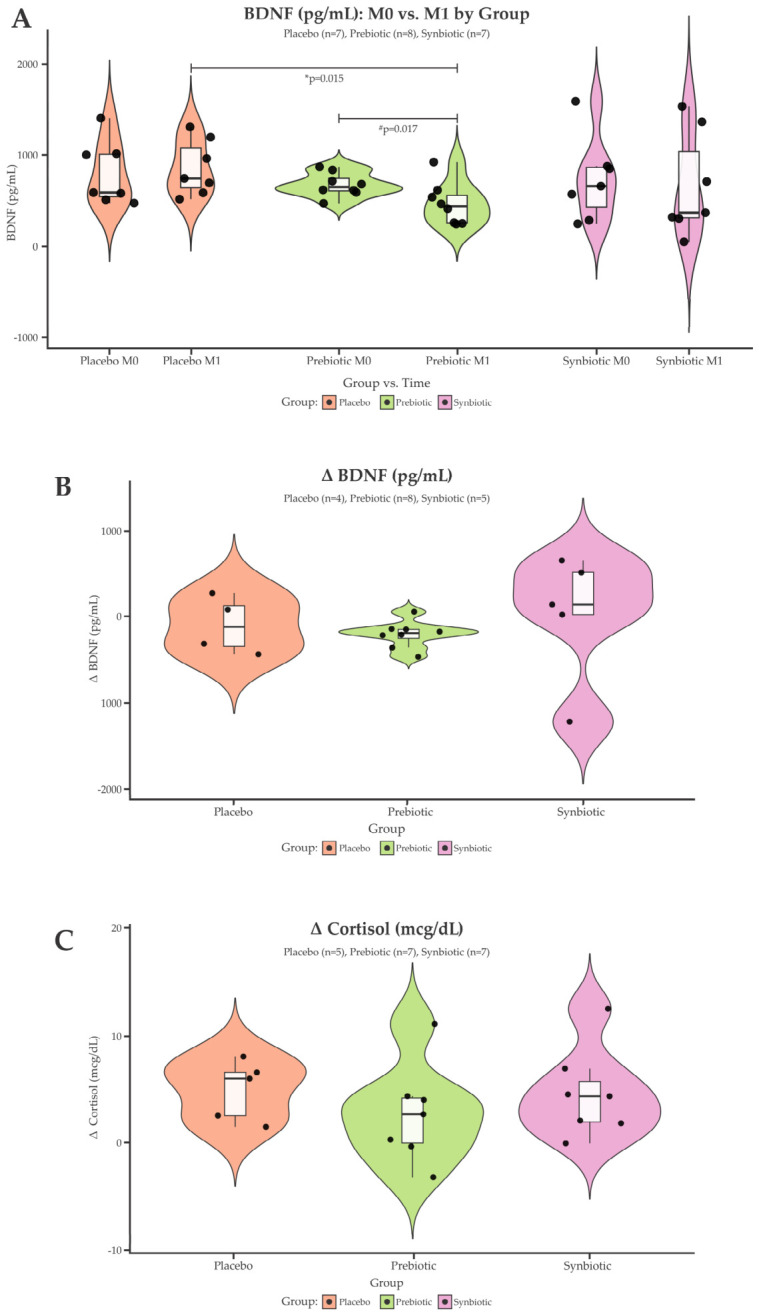
Serum BDNF and Cortisol concentrations across experimental groups. (**A**): Serum BDNF concentrations observed across experimental groups at baseline and after 30 days of supplementation. (**B**): Changes in serum BDNF concentrations between the baseline and endline, observed among individuals without an anxiety diagnosis. (**C**): Changes in serum cortisol concentrations between the baseline and endline, observed among subjects without a diagnosis of depression. Concentrations are expressed as median and interquartile range. * Mann–Whitney U test. # Wilcoxon signed-rank test for paired data. Violin plots display Kernel Density Estimation (KDE) with individual data points and median/IQR indicated by boxplots.

Serum BDNF concentrations decreased significantly after 30 days of prebiotic supplementation (*p* = 0.017; Figure 2A); when the comparison of the change between the baseline and endline was analyzed, only this group showed a reduction in this parameter (Table 2). In the intergroup analysis after 30 days of intervention, the prebiotic group had significantly lower serum BDNF concentrations than the placebo group (*p* = 0.015; Figure 2A). Regarding the difference between the baseline and endline and performing an analysis excluding subjects who were diagnosed with anxiety revealed that the group receiving synbiotic supplementation was the only one to show an increase in serum BDNF concentration, although this did not reach statistical significance (Figure 2B).

For the sole purpose of illustrating the detectable threshold, we calculated the minimum detectable difference (MDD) of BDNF (primary outcome of this paper), considering an alpha (α) value of 5%, beta (β) value of 20%, the sample size obtained at the end of the study (7 individuals per group) and a standard deviation calculated from the 95% CI = 6.62 ng/mL, observed in the meta-analysis by Molendijik et al. [28]. Thus, we identified that the MDD (*p* < 0.05) for BDNF, with the sample obtained in this study is 9.92 ng/mL, assuming the standard deviation is equal to or less than 6.62 ng/mL.

The data presented in Table 2 show that serum cortisol concentrations increased in all groups between baseline and endline, reaching statistically significant in the synbiotic-supplemented group (*p* = 0.028). Regarding the difference between the baseline and endline and performing an analysis excluding subjects diagnosed with depression revealed that the group receiving prebiotic supplementation had a smaller increase in serum cortisol concentrations, although this was not statistically significant (Figure 2C).

The data presented in Table 2 also show that serum TNF-α concentrations increased significantly after 30 days of prebiotic supplementation when compared to the group’s baseline (*p* = 0.035). However, this variation did not produce a significant difference between the groups evaluated after 30 days of supplementation. For the other variables, no significant differences were found between groups or within the same group between baseline and post-intervention (Table 2).

Correlation analyses revealed mostly weak and nonsignificant associations among biomarkers. Notably, a very strong positive correlation (r = 0.892; *p* = 0.006) was observed between serum BDNF and vitamin B12 concentrations, as well as a strong negative correlation—though not statistically significant (r = −0.714; *p* = 0.071)—between serum BDNF and IL-6 concentrations in subjects who received synbiotic supplementation for 30 days (Table 3). Among placebo-treated subjects, we observed a strong positive correlation between serum ACTH and IL-6 concentrations (r = 0.785; *p* = 0.032). These effects were not observed with the other treatments (placebo or prebiotic), as shown in Table 3. Given the very small sample size within each experimental group, these correlation analyses are highly exploratory, potentially unstable, and must be interpreted with extreme caution, serving exclusively as preliminary hypothesis-generating signals.

## 4. Discussion

The association between obesity and neuroendocrine changes related to diagnoses of depression and anxiety reported in the literature [1,4,29,30], was consistent with the proportion of subjects diagnosed with depression and/or anxiety (31.8%; *n* = 7) and the continuous use of antidepressants (fluoxetine, sertraline, bupropion or clonazepam) (31.8%; *n* = 7) observed in our sample. Interestingly, two subjects diagnosed with depression and/or anxiety were not taking antidepressants, while two subjects who used these drugs (prescribed in the medical record) had no diagnosis of an emotional disorder. These findings highlight the difficulty of establishing a standardized diagnosis for these emotional disorders, especially in the presence of other neuroendocrine disorders, such as obesity [31,32,33,34].

The relationship of depression and anxiety with obesity is mainly related to common endocrine changes, such as HPA axis hyperactivity [1,3,9], characterized by ACTH and cortisol hypersecretion [2,3,4]. In our study, serum ACTH concentrations were within the reference ranges for all samples analyzed and did not change significantly after the interventions. In contrast, cortisol concentrations, although also within the reference ranges, increased in all groups, with a significant difference between baseline and endline in the synbiotic group. Importantly, when evaluating only individuals without a diagnosis of depression, those who received prebiotic supplementation showed the lowest cortisol increase.

Schachter et al. [1], in a literature review, they observed that neurochemical changes and the maintenance of a chronic inflammatory status are related to high-fat diets and obesity, in addition to correlating with increased emotional disorders, such as depression and anxiety, especially in experimental studies [35,36,37,38,39,40]. The authors of the review [1] point out that the use of probiotics and prebiotics to maintain the integrity of the intestinal microbiota could be used to prevent and treat obesity and emotional disorders. In an experimental model (mice), supplementation with combined FOS and GOS was more effective than supplementation with these prebiotics in isolation in reducing serum corticosterone concentrations and increasing the gene expression of hippocampal BDNF [40].

In our clinical trial, it was observed that all subjects, both before and after any of the interventions (placebo, prebiotic, or synbiotic), had serum BDNF concentrations below the reference ranges. Prebiotic treatment for 30 days further reduced these levels, while placebo and synbiotic treatment for the same period promoted only slight changes that did not reach statistical significance. It is possible that in individuals with morbid obesity, the microbiome response differs from that of the general population due to the well-established chronic low-grade inflammatory state [1]. Furthermore, the intervention period (30 days), although justified by systematic review evidence [22], may have been insufficient to overcome the initial adaptation or metabolic stress phase. It is important to note that our sample was not large enough to ensure sufficient statistical power to identify differences if they exist. However, considering the calculation of the MDD for BDNF—based on a meta-analysis published in 2014 with an evaluation of 5203 individuals [28]—the variations observed in our sample would be significant if our standard deviation were smaller. These findings lead us to reflect on the statistical significance of variations in BDNF concentrations in this population. Nevertheless, the marked gender imbalance across groups (Placebo: 2M/5F; Prebiotic: 0M/8F; Synbiotic: 4M/3F; *p* = 0.057; Table 1) constitutes a critical limitation, particularly in the interpretation of these results. Sex-based differences are well-established in BDNF regulation, HPA axis responses to microbiota modulation, and gut–brain axis signaling [41,42,43]. This imbalance likely amplified between-group variability and precludes sex-specific mechanistic interpretations. Sex-stratified analyses were not conducted due to insufficient power.

Additionally, serum TSH, vitamin B12, and folic acid concentrations were within the reference ranges and were not altered by the interventions. However, a positive correlation was observed between serum BDNF and vitamin B12 concentrations after 30 days of synbiotic supplementation, suggesting that the synbiotic may optimize nutrient absorption (B12) which, in turn, supports neuronal health (BDNF), even though absolute levels did not reach statistical significance. Experimental studies in mice have shown that the administration of prebiotics (FOS and/or GOS) [35] or probiotics [44] promotes increased BDNF expression and reduces anxiety- and depression-like behaviors. Neuroendocrine communication is dependent on several metabolic factors, such as the synthesis and release of neurotransmitters and hormones and the expression of receptors and ion channels, all of which are influenced by membrane permeability and cellular integrity. These reactions are largely dependent on B vitamins; therefore, their deficiency is related to clinical symptoms of depression and anxiety and reduced cognitive function [5,6,45].

In depression and anxiety, hormonal changes that affect the HPA axis also promote parathyroid gland stimulation with the consequent increase in PTH concentrations [8], which is accompanied and intensified by a reduction in vitamin D concentrations [46]. In our clinical trial, baseline PTH serum concentrations were above reference ranges in the prebiotic and synbiotic-supplemented groups. After 30 days of prebiotic supplementation this parameter normalized, while the symbiotic-supplemented group presented a mean increase of 11.7 pg/mL. Serum vitamin D concentrations were below the reference ranges in the placebo group only, with this deficit increasing after 30 days of supplementation. This change did not influence serum PTH concentrations, which remained within the reference range in this group. Prebiotic or symbiotic-supplemented groups started with serum vitamin D concentrations within the reference ranges and showed an increase after 30 days of intervention, most notably in the synbiotic group. Thus, the results of our clinical trial show that prebiotic or synbiotic supplementation improved serum vitamin D concentrations; however, the inverse relationship with PTH was only observed in the prebiotic group and did not reach statistical significance. A supplementation period longer than 30 days may be necessary to demonstrate a sustained response pattern to prebiotic or synbiotic supplementation.

Studies show that serum PTH and vitamin D concentrations are inversely related and that in depression and/or anxiety there is an increase in serum PTH concentrations alongside a reduction in vitamin D [7,8]. Studies in elderly individuals with depression have observed serum PTH concentrations similar to those of healthy elderly control, while vitamin D concentrations were lower among the elderly with depression when compared to the group without depression [47]. Vitamin D deficiency, commonly observed in obesity and associated with low physical activity, poor nutrition, and chronic inflammation, is among the risk factors for the development of emotional disorders such as depression and anxiety [13].

Communication along the gut–brain axis participates in the regulation of the synthesis and secretion of hormones, neurotransmitters, and cytokines [17,18,44,48], suggesting that maintaining the integrity of the intestinal microbiota may act as a therapeutic adjunct for emotional disorders [48]. Dysbiosis is associated with chronic inflammation, reduced serum BDNF concentrations, impaired memory and increased anxiety and depression in humans [11,49]. On the other hand, probiotic supplementation has been shown to reduce corticosterone, adrenaline and noradrenaline concentrations in mice under chronic stress [44].

Bruce-Keller, Salbaum, and Berthoud [12] showed in a review that the administration of prebiotics, probiotics, and fecal microbiota transplantation has beneficial effects in reducing symptoms associated with emotional disorders, as evidenced in experimental [44,50,51,52] and clinical studies [53,54,55,56]. However, the authors of the review [12] warn that the exact mechanisms by which such benefits are obtained are not yet fully understood, and further well-designed and controlled studies are needed to identify the pathways and markers involved.

In our clinical trial, where the sample consisted of individuals with morbid obesity, they presented a low-grade chronic inflammatory state, evidenced by serum hs-CRP, IL-1β and IL-6 concentrations above the reference ranges for healthy individuals both at baseline and after 30 days of intervention. Serum TNF-α concentrations, which although significantly increased after 30 days of prebiotic supplementation, remained within the reference range at both time points analyzed for all experimental groups. It is important to highlight that the reduction in serum BDNF concentrations, accompanied by increased TNF-α in individuals receiving prebiotics, unexpectedly signals that the intervention may elicit a pro-inflammatory response in this specific population (women with morbid obesity). Studies with adequate statistical power and gender-stratified analyses are needed to test this hypothesis. Additionally, a strong negative correlation—though not statistically significant—was observed between BDNF and IL-6 in the synbiotic group and a strong positive correlation between ACTH and IL-6 in the placebo group, illustrating the relationship between low-grade chronic inflammation and neuroendocrine changes in this population. However, given the very small sample size in each group, these findings should be interpreted cautiously and considered exploratory.

A meta-analysis by McLoughlin et al. [57] showed that supplementation with prebiotics or synbiotics promoted a reduction in serum CRP concentrations. However, the authors reported that only 48% of studies using prebiotic supplementation showed a reduction in inflammatory parameters, similar to the findings of a systematic review conducted by our research group involving overweight or obese individuals [22]. It is believed that the heterogeneity of experimental designs, as well as differences in dosage and intervention duration, are among the possible causes of these divergent results [22,57].

In summary, our clinical study did not demonstrate the expected biochemical effects of prebiotic or synbiotic supplementation. Our main limitation was the small sample size at the end of the experimental period, resulting from stringent inclusion and retention criteria. These criteria were designed to obtain a homogeneous sample free from confounding factors influencing the gut microbiota. Unfortunately, this led to a small sample from the outset, and we were further affected by a follow-up loss of 42% (*n* = 16) within 30 days of treatment, greatly limiting the power of our results. The true voluntary dropout rate was 21% (8/38), with scheduling conflicts (no adverse events confirmed) as the primary driver in this working-age population with obesity. Protocol deviations (21%) reflect methodological rigor in maintaining internal validity despite external challenges. The absence of significant results in the primary outcomes is possibly due to the study being severely underpowered; thus, findings from additional secondary outcomes are hypothesis-generating only, requiring confirmation in adequately powered trials. Additionally, we found differences in the gender distribution between the study groups, which, although not significant, may have contributed to the variability of the results and, consequently, to the respective standard deviations. Sex-stratified analyses were not performed due to the insufficient sample size, precluding meaningful subgroup power. This limitation prevents sex-specific mechanistic interpretations and underscores the need for sex-balanced confirmatory trials. Future studies should employ stratified block randomization to address this critical methodological gap.

We also emphasize that this short-term feasibility RCT (30 days, consistent with systematic review standards) presents hypothesis-generating results that justify investigation in long-term trials (≥90 days) with adequate statistical power. Additionally, the evaluation of neuroendocrine interactions related to emotional disorders was incorporated into the research while it was in progress; consequently, it was not possible to apply any tool for the assessment of emotional disorders or to maintain balance across groups regarding diagnoses and pharmacological treatment. Therefore, we lack sufficient information to correlate the biochemical results found with clinical parameters related to emotional disorders. However, our clinical study was carried out following rigorous methods to mitigate factors that could influence the composition of the gut microbiota, leading us to believe that the effects of prebiotic and synbiotic supplementation could be statistically observed with a supplementation period longer than 30 days.

## 5. Conclusions

In conclusion, the biomarker changes observed in individuals with morbid obesity support the hypothesis that their chronic low-grade inflammation may be associated with the neuroendocrine alterations seen in emotional disorders such as depression and anxiety. In this context, maintaining healthy gut microbiota may contribute to the regulation of biochemical balance through the gut–brain axis. Despite significant limitations—including limited statistical power, a lack of sex stratification, and the absence of clinical indicators of emotional disorders—these biomarker changes justify long-term clinical trials with adequate statistical power, sex-balance, validated clinical scales, microbiome sequencing, and multi-omic analyses.

## Figures and Tables

**Table 1:** Baseline characteristics of the study participants.

Characteristics	Study Groups			*p* Value
	Placebo (*n* = 7)	Prebiotic (*n* = 8)	Synbiotic (*n* = 7)	
Age (years)	43.9 ± 10.0	40.5 ± 10.3	41.3 ± 4.9	0.753 ^a^
Gender (Male/Female)	2/5	0/8	4/3	0.057 ^b^
Body weight (kg)	135.3 ± 28.4	119.9 ± 17.9	129.0 ± 25.4	0.473 ^a^
Body Mass Index (kg/m^2^)	50.9 ± 9.4	46.6 ± 5.3	46.0 ± 5.2	0.363 ^a^
Waist circumference (cm)	137.3 ± 13.1	126.3 ± 11.6	136.1 ± 14.4	0.216 ^a^
Functional constipation (*n*/%) *	0 (0)	2 (25.0)	0 (0)	0.582 ^b^
Stool consistency and shape (Bristol Scale)				1.000 ^b^
Types 1–3 or 5–7	5 (71.4)	5 (75.0)	5 (71.4)	
Type 4	2 (28.6)	2 (25.0)	2 (28.6)	
Menopause (*n*/%)	2 (28.6)	2 (25.0)	0 (0)	0.582 ^b^
Associated comorbidities (*n*/%)				
Systemic arterial hypertension	4 (57.1)	1 (12.5)	3 (42.9)	0.119 ^b^
Anxiety/Depression	4 (57.1)	1 (12.5)	2 (28.6)	0.282 ^b^
Type 2 Diabetes Mellitus	1 (14.3)	0 (0)	2 (28.6)	0.303 ^b^
Dyslipidemia	1 (14.3)	0 (0)	1 (14.3)	0.500 ^b^
Others **	1 (14.3)	3 (37.5)	4 (57.1)	0.266 ^b^
Drugs for continuous use (*n*/%)				
Antihypertensives	4 (57.1)	1 (12.5)	3 (42.9)	0.119 ^b^
Antidepressants	4 (57.1)	1 (12.5)	2 (28.6)	0.282 ^b^
Oral hypoglycemic agents	1 (14.3)	0 (0)	2 (28.6)	0.303 ^b^
Statins	0 (0)	0 (0)	0 (0)	1.000 ^b^
Others ***	1 (14.3)	2 (25.0)	1 (14.3)	1.000 ^b^

Continuous variables are expressed as mean and standard deviation. ^a^ One-way ANOVA test. ^b^ Fisher’s exact test. * According to the Rome IV criteria (Lacy et al.) [26]. ** Psoriasis, hypothyroidism, gastroesophageal reflux, sleep apnea, panic syndrome, congestive heart failure, and varicose veins in the lower limbs. *** Antacids, thyroid hormones and venotonics.

**Table 2:** Biochemical parameters analyzed across experimental groups.

Outcomes	Study Groups			*p* Values ^a^
	Placebo	Prebiotic	Synbiotic	
BDNF (pg/mL)	(*n* = 7)	(*n* = 8)	(*n* = 7)	
Baseline	575.2 (496.6; 1001.7)	635.8 (588.3; 761.7)	645.3 (271.1; 864.7)	0.959
Endline	731.2 (572.8; 1182.7)	424.7 (241.0; 563.0)	355.2 (291.9; 1350.9)	0.098
*p* value (paired test) ^b^	0.735	0.017	0.735	
Difference between moments	76.16 (−319.9; 305.2)	−194.6 (−291.5; −148.3)	71.9 (−603.2; 514.2)	0.226
ACTH (pg/mL)	(*n* = 7)	(*n* = 8)	(*n* = 7)	
Baseline	13.9 (9.5; 17.2)	12.7 (11.2; 15.0)	19.0 (7.5; 35.6)	0.759
Endline	12.5 (7.9; 15.9)	17.6 (12.6; 19.3)	21.5 (7.0; 42.6)	0.368
*p* value (paired test) ^b^	0.671	0.092	0.612	
Difference between moments	−0.7 (−3.8; 3.0)	3.3 (1.0; 7.1)	1.2 (−4.5; 9.6)	0.343
Cortisol (µg/dL)	(*n* = 7)	(*n* = 8)	(*n* = 7)	
Baseline	9.4 (4.8; 11.7)	7.7 (5.5; 9.3)	6.2 (5.7; 7.3)	0.223
Endline	10.8 (8.4; 12.4)	9.6 (6.9; 11.2)	10.2 (8.6; 12.9)	0.751
*p* value (paired test) ^b^	0.398	0.092	0.028	
Difference between moments	2.5 (−4.7; 6.5)	3.3 (−0.0; 4.1)	4.3 (1.8; 6.9)	0.678
TSH (µUI/mL)	(*n* = 6)	(*n* = 7)	(*n* = 7)	
Baseline	2.34 (1.6; 2.4)	2.08 (1.7; 3.2)	1.56 (1.1; 2.3)	0.375
Endline	2.14 (1.8; 2.9)	2.52 (1.7; 4.1)	2.12 (1.8; 2.5)	0.422
*p* value (paired test) ^b^	0.600	0.236	0.310	
Difference between moments	0.09 (−0.1; 0.2)	0.45 (−0.4; 1.2)	0.18 (−0.07; −0.5)	0.710
PTH (pg/mL)	(*n* = 6)	(*n* = 8)	(*n* = 7)	
Baseline	56.3 (45.5; 62.3)	67.1 (61.5; 108.0)	82.8 (48.7; 86.6)	0.212
Endline	51.2 (27.4; 64.4)	64.0 (58.2; 79.2)	80.8 (46.0; 98.8)	0.141
*p* value (paired test) ^b^	0.173	0.123	0.398	
Difference between moments	−8.9 (−14.9; 2.9)	−5.95 (−15.9; 1.7)	11.7 (−5.2; 14.5)	0.201
Vitamin D (ng/mL)	(*n* = 7)	(*n* = 8)	(*n* = 7)	
Baseline	19.8 (13.7; 23.1)	23.0 (15.9; 25.1)	25.2 (14.8; 31.0)	0.362
Endline	19.3 (11.7; 23.8)	23.2 (19.7; 25.3)	27.6 (24.0; 33.5)	0.079
*p* value (paired test) ^b^	0.865	0.327	0.120	
Difference between moments	−1.3 (−2.9; 4.0)	0.7 (−1.0; 2.8)	2.5 (−0.7; 2.8)	0.429
Vitamin B12 (pg/mL)	(*n* = 7)	(*n* = 8)	(*n* = 7)	
Baseline	419.0 (298.0; 627.0)	348.0 (289.5; 446.5)	546.0 (345.0; 603.0)	0.338
Endline	325.0 (309.0; 588.0)	347.0 (289.0; 431)	516.0 (375.0; 610.0)	0.247
*p* value (paired test) ^b^	0.865	0.888	0.671	
Difference between moments	8.0 (−76.0; 105.0)	4.0 (−93.5; 57.5)	28.0 (−30.0; 48.0)	0.918
Folic acid (ng/mL)	(*n* = 7)	(*n* = 8)	(*n* = 7)	
Baseline	11.8 (9.1; 21.7)	13.1 (10.2; 14.2)	15.7 (11.6; 24.1)	0.323
Endline	11.5 (7.8; 15.1)	14.1 (7.9; 16.3)	12.9 (11.8; 16.4)	0.700
*p* value (paired test) ^b^	0.499	0.674	0.176	
Difference between moments	−1.8 (−8.6; 5.1)	1.0 (−3.0; 4.9)	−3.1 (−7.6; 2.1)	0.408
*hs*-CRP (mg/L)	(*n* = 7)	(*n* = 8)	(*n* = 7)	
Baseline	8.9 (3.5; 10.5)	11.2 (7.9; 19.8)	9.0 (2.5; 10.8)	0.339
Endline	5.8 (5.5; 22.8)	8.6 (4.1; 13.4)	6.7 (4.1; 10.5)	0.957
*p* value (paired test) ^b^	0.671	0.207	0.735	
Difference between moments	−1.2 (−3.2; 2.9)	−1.9 (−5.5; 1.3)	1.5 (−3.1; 1.6)	0.606
IL-1β (pg/mL)	(*n* = 7)	(*n* = 8)	(*n* = 7)	
Baseline	7.7 (2.2; 8.1)	6.5 (5.5; 7.7)	3.0 (0; 9.5)	0.502
Endline	7.9 (6.6; 10.0)	8.2 (6.3; 10.7)	5.9 (0; 7.2)	0.097
*p* value (paired test) ^b^	0.310	0.262	0.799	
Difference between moments	1.9 (−2.2; 7.0)	1.6 (−0.7; 4.1)	0 (−3.6; 3.2)	0.547
IL-6 (pg/mL)	(*n* = 7)	(*n* = 8)	(*n* = 7)	
Baseline	8.7 (6.2; 11.2)	10.7 (8.5; 12.4)	6.6 (4.5; 10.5)	0.103
Endline	9.8 (5.6; 12.2)	11.3 (6.7; 12.0)	7.7 (4.6; 9.1)	0.495
*p* value (paired test) ^b^	0.499	0.483	0.612	
Difference between moments	1.1 (−1.1; 2.6)	−0.7 (−1.8; 1.2)	0.09 (−1.4; 2.5)	0.510
TNF-α (pg/mL)	(*n* = 7)	(*n* = 8)	(*n* = 7)	
Baseline	4.0 (2.6; 8.6)	4.4 (3.4; 5.8)	4.9 (0; 10.7)	0.962
Endline	5.0 (1.9; 13.8)	7.0 (3.6; 11.5)	6.7 (3.6; 11.8)	0.927
*p* value (paired test) ^b^	0.735	0.035	0.350	
Difference between moments	0.7 (−2.6; 5.5)	2.9 (0.4; 5.3)	1.3 (−1.6; 6.8)	0.645

Continuous variables are expressed as median and interquartile range. ^a^ Kruskal–Wallis test. ^b^ Wilcoxon signed-rank test for paired data. The difference between time points was calculated as: endline value (30 days) minus baseline value. Nominal *p*-values (uncorrected for multiple testing) are provided from the exploratory biomarker analysis. Type I error risk is explicitly acknowledged.

**Table 3:** Neuroendocrine and inflammatory correlations across experimental groups after 30 days of supplementation.

Groups	BDNF vs. Vit. B12		BDNF vs. IL-6		BDNF vs. ACTH		ACTH vs. IL-6	
	r-Values	*p*-Values	r-Values	*p*-Values	r-Values	*p*-Values	r-Values	*p*-Values
Placebo	−0.0357	0.9394	−0.2143	0.6445	−0.6071	0.1482	0.7857	0.0362
Prebiotic	−0.0476	0.9108	0.0952	0.8225	−0.3095	0.4556	0.3095	0.4556
Synbiotic	0.8929	0.0068	−0.7143	0.0713	−0.3571	0.4316	−0.0714	0.8790

The “r” values were obtained using Spearman correlation. Note: Due to the small sample size (*n* = 7–8 per group), these correlation coefficients are exploratory and highly susceptible to individual variability. Nominal *p*-values are reported without correction for multiple testing, and these preliminary associations require validation in adequately powered confirmatory trials.

## Data Availability

The original data presented in the study are openly available in OSF as a public project ID: Bh6ud, available at: https://osf.io/bh6ud/overview, accessed on 22 April 2026.

## Supplementary Materials

The following supporting information can be downloaded at: https://www.mdpi.com/article/10.3390/life16060924/s1, File S1: CONSORT checklist.
